# The value of real world evidence: The case of medical cannabis

**DOI:** 10.3389/fpsyt.2022.1027159

**Published:** 2022-11-03

**Authors:** Anne Katrin Schlag, Rayyan R. Zafar, Michael T. Lynskey, Alkyoni Athanasiou-Fragkouli, Lawrence D. Phillips, David J. Nutt

**Affiliations:** ^1^Drug Science, London, United Kingdom; ^2^Department of Brain Sciences, Faculty of Medicine, Imperial College London, London, United Kingdom; ^3^Department of Management, London School of Economics and Political Science, London, United Kingdom

**Keywords:** medical cannabis, cannabis based medicinal products (CBMPs), real world evidence (RWE), patient reported outcomes (PROs), patient access

## Abstract

Randomised controlled trials (RCTs) have long been considered the gold standard of medical evidence. In relation to cannabis based medicinal products (CBMPs), this focus on RCTs has led to very restrictive guidelines in the UK, which are limiting patient access. There is general agreement that RCT evidence in relation to CBPMs is insufficient at present. As well as commercial reasons, a major problem is that RCTs do not lend themselves well to the study of whole plant medicines. One solution to this challenge is the use of real world evidence (RWE) with patient reported outcomes (PROs) to widen the evidence base. Such data increasingly highlights the positive impact medical cannabis can have on patients’ lives. This paper outlines the value of this approach which involves the study of interventions and patients longitudinally under medical care. In relation to CBMPs, RWE has a broad range of advantages. These include the study of larger groups of patients, the use of a broader range and ratio of components of CBMPs, and the inclusion of more and rarer medical conditions. Importantly, and in contrast to RCTs, patients with significant comorbidities–and from a wider demographic profile–can also be studied, so providing higher ecological validity and increasing patient numbers, whilst offering significant cost savings. We conclude by outlining 12 key recommendations of the value of RWE in relation to medical cannabis. We hope that this paper will help policymakers and prescribers understand the importance of RWE in relation to medical cannabis and help them develop approaches to overcome the current situation which is detrimental to patients.

## Introduction: Medical cannabis in the United Kingdom

Cannabis is one the oldest medicines known, having been used for millennia to treat a broad range of health conditions (1). In the UK, medical cannabis was legalised on 1st November 2018 and made available under a legislative change as a result of public controversy and campaigning. The law adjusting the Misuse of Drugs Regulations 2001 was The Misuse of Drugs (Amendments) (Cannabis and Licence Fees) (England, Wales, and Scotland) Regulations 2018.

As early as 1998, a House of Lords report on medical cannabis provided clear evidence on the efficacy and value of CBMPs (2). The whole plant extract mixture of delta-9-tetrahydrocannabinol (THC, also known as dronabinol) and cannabidiol (as Sativex) has been a licenced medicine in the UK for over a decade. The decision to move cannabis to Schedule 2 nearly 4 years ago was made on the basis that there were adequate data that it was a medicine (3). Nevertheless, since 2018 only three people (all children) have been prescribed with a full spectrum product within the UK National Health Service (NHS). [NB: As Sativex is a whole plant extract containing both THC and CBD, the same drugs are listed in two sections of the scheduling, hence the need for Sativex to be listed by brand name and to be placed in a different schedule. “Sativex” is Schedule 4].

The current UK National Institute for Health and Care Excellence (NICE) guidelines recommend the prescription of two cannabis-based medicinal products (CBMPs) and one synthetic cannabinoid with five licenced indications. Sativex is licenced for spasticity in Multiple Sclerosis (MS), Epidyolex is licenced for seizures in tuberous sclerosis complex, Lennox-Gastaut and Dravets syndromes, and nabilone (a synthetic THC mimic) is licenced for chemotherapy induced nausea. Dronabinol (synthetic d9THC) is prescribed for a wide range of indications in the USA but does not have marketing authorisation in the UK.

These NICE guidelines- as well as guidelines by other institutions, such as the British Paediatric Neurology Association (BPNA)- are based largely on evidence collated through randomised controlled trials (RCTs), neglecting patient reported outcomes (PROs). As such, they have been criticised by patients, campaigners, and some doctors as too limiting. In contrast to the UK, in many other countries, a broad range of cannabis based medicinal products (CBMPs) have been made available to patients in a short space of time (4).

As licenced medications, both Epidyolex and Sativex can be prescribed off label for any condition a physician feels they may benefit. In principle, this presents a distinct advantage compared with other CBMPs under the “specials” prescribing system. In practice however, NHS funding is only available for their approved indications. The underlying reason for this restriction in funding is the lack of RCT evidence for cost effectiveness that is required by NICE to justify the NHS paying for medicines.

Furthermore, the medical and related professions (especially pharmacy) have come to believe that NICE advice is a directive, rather than a guidance. The lack of guidance on the unlicenced use of CBMPs therefore dissuades many health care providers from considering their use and supporting their patients’ use of cannabis. Access to CBMPs *via* the private sector is an option but prevents access to those without the necessary finances.

Wider access to CBMPs on the NHS may well have the potential to be a cost saving measure in the longer term by reducing the reliance on more expensive medications- if the price and availability of CBMPs can be reduced. Some examples here might include the reduction of the use of biologics and monoclonal antibodies in autoimmune diseases like rheumatoid or Crohn’s disease, whereby CBMPs would likely be cheaper in this instance. In order to assess the cost effectiveness of CBMPs for various conditions, health economic analyses are required (5).

The therapeutic potential of CBMPs to treat a variety of conditions is becoming increasingly recognised (6). Globally, a growing number of countries have now legalised cannabis for medical uses and a substantial number of patients are able to access their medications (7). Despite international evidence and even though a considerable number of patients are already treating a broad range of conditions with CBMPs, the focus on RCTs and consequently, licencing issues, the limitation of products and indications, the costs of private prescriptions as well as physician hesitancy mean that patient access in the UK remains very restricted. In order to develop the current evidence base, the importance of incorporating real-world data (RWD) to assess the clinical effectiveness of medical cannabis is gradually becoming recognised.

## Limitations of randomised controlled trials

Placebo-controlled double-blind or randomised controlled trials (RCTs) have been a key element of medical drug development, with the ultimate goal of providing evidence for pharmaceutical drug development companies to gain marketing authorisation (i.e., a license to sell) for their investigational medicinal products. RCTs have dominated the last 50 years of drug development and as a result have become considered the “gold standard” for all medical evidence. Consequently, until recently, this has precluded other approaches such as real world trials, which may have as much- if not more- utility in relation to some types of drug, including medical cannabis.

The perception that RCTs are needed before any conclusions on effectiveness can be proved is a common misunderstanding of the nature of medical evidence (8). Although care should be taken when comparing clinical responses without head-to-head comparisons (owing to differences in study design, population, and so on), Sir Michael Rawlins, the former head of the MHRA and NICE, pointed out in 2008 that RCTs are not the apex of treatment trials:

*“Randomised controlled trials, long regarded at the “gold standard” of evidence, have been put on an undeserved pedestal. Their appearance at the top of “hierarchies” of evidence is inappropriate; and hierarchies, themselves, are illusory tools for assessing evidence. They should be replaced by a diversity of approaches that involve analysing the totality of the evidence base”* (9).

These issues with RCTs are described in detail below.

### Lack of ecological validity

Randomised controlled trials are undertaken in tightly selected patient groups that are not usually representative of the average patient who often presents with multiple medical comorbidities and who is also prescribed other medicines. Exclusion of these individuals means that safety data derived from RCTs often lacks ecological validity. Their data may be of low relevance to their use in normal medical settings where comorbidities and drug-drug interactions which have been actively excluded from the study by design may pose uncertain health risks.

Therefore, even when such trials are positive, they are only suggestive of effectiveness in the wider patient groups. RCTs measure the efficacy of a treatment but this does not equal effectiveness. Efficacy is based on the change in clinical endpoints at defined time-points and these are pre-registered within the construct of a controlled clinical trial. Effectiveness assesses the utility of the medicine in the real world, and assesses changes in patient health outcomes that extend beyond pre-defined clinical scales or time points. Approaches such as effectiveness trials or clinical audits are often required to best estimate the real-world value of an intervention to individual patients. These usually take place in Phase IV of the clinical trial drug development pipeline during a “pharmacovigilance post-marketing phase”. Adoption of such an approach to unlicensed medicines such as medical cannabis, prior to initiation of clinical trials, however, could be fruitful in providing data on the effectiveness of an intervention. Such data could subsequently guide the better development of controlled clinical research programs.

### Prohibitive costs and reliance upon patentable returns

Randomised controlled trials are time consuming and often prohibitively expensive and, with new medicines, largely conducted by for-profit pharmaceutical companies. Very few of the cannabis- responsive conditions reported by patients are currently being studied using RCTs. Reasons for this include difficulties in patenting whole plant extracts given their historical use and complex mixture of cannabinoids, terpenes, and flavonoids, issues related to reproducibility between crops, and the reluctance of the UK to licence plant-based medicines (please see Box 1 on botanical medicines).

**BOX 1. Botanical medicines.**

Botanical medicines are defined by the Medicines and Healthcare Regulatory Agency (MHRA) as:

*“a product is a herbal medicinal product if the active ingredients are herbal substances and/or herbal preparations only.*

***An herbal substance** is a plant or part of a plant, algae, fungi or lichen, or an unprocessed exudate of a plant, defined by the plant part used and the botanical name of the plant, either fresh or dried, but otherwise unprocessed.*

***An herbal preparation** is when herbal substances are put through specific processes, which include: extraction, distillation, expression, fractionation, purification, concentration, fermentation.*

*The herbal substance being processed can be: reduced or powdered, a tincture, an extract, an essential oil, an expressed juice, a processed exudate (rich protein oozed out of its source).”* (https://www.gov.uk/guidance/apply-for-a-traditional-herbal-registration-thr#herbal-medicinal-product-definition)

The traditional RCT approach was used for cannabidiol (CBD) in two forms of childhood epilepsy (Lennox-Gastaut and Dravet syndromes) by GW Pharmaceuticals. The clinical trials took nearly 20 years to complete, and the company’s application for NHS use was then turned down by NICE on the grounds of cost-effectiveness, although this has now been reversed (10). Unsurprisingly, other companies have seen this as a serious barrier to moving into this field. If the same requirement for RCT evidence had been applied to penicillin, it might never have been developed as a medicine (8).

Randomised controlled trials in contrast can provide data at a relatively low cost compared with RCTs so can engage much larger numbers of patients. This gives them a great advantage in terms of estimates of uncommon adverse effects.

### Limited long term patient safety data

Another argument for RCT testing is to measure the safety of compounds to discover potentially serious adverse effects such as observed with thalidomide. However, neither RCTs nor pre-clinical toxicology and safety studies can mitigate such concerns. For example, in the case of thalidomide, pre-clinical testing would not have detected the teratogenesis as it did not cause malformations in rodents and RCTs almost always exclude pregnant women. Cannabis has been used as a medicine for millennia, and with tens of millions of recreational users internationally, many of whom are women, no significant foetal harm has been reported in the medical or scientific literature (8). One of the key arguments made against prescribing medical cannabis- that it could be harmful because of a lack of traditional safety testing *via* RCTs–is therefore unsound. The reality is that thousands of years of real world evidence on the safety of cannabis compounds is being overlooked (8).

Given these limitations of RCTs, there are many other forms of evidence that can equally inform medical practice. These include RWE such as pharmacoepidemiology, patient-reported outcomes, effectiveness trials, and case series, which we outline in the following sections. Many of these require unlicenced or off-licenced prescribing which we briefly detail here.

### Prescribing unlicensed medicines

Even with positive efficacy data and proven safety profile for a concrete indication in a pure patient population, the approval process applied to medicines does not guarantee successful treatment for every patient. Patients often go through a long journey of a repetitive trial and error approaches of different medicines without a clinically meaningful response. As part of this process, the totality of available literature, from patient reported outcomes, twinned with trial data should be utilised by clinicians in deciding clinical prescribing regimens.

Regarding concerns about prescribing unlicensed medicines, there is less protection for the clinician for unlicenced prescribing. This leads to many doctors being hesitant to prescribe, especially when a controlled drug is used. Additionally, as CBMPs fall under the specials scheme this adds complexity to sourcing and dispensing. Apart from nabilone, Sativex, and Epidyolex, all CBMPs in the UK are unlicenced and therefore carry perceived risks to the prescriber, which may present a barrier to those without the appropriate knowledge. By gathering RWE from projects such as Project Twenty21 (T21) some of this anxiety can be mitigated as it may help guide doctors with insufficient knowledge of CBMPs as to when it might be appropriate to prescribe.

Clinicians should leverage publicly available resources as well as communicating to other prescribing clinicians to guide their own practice where they deem it to be in the patients’ best interests. As of late, several clinical audits are becoming available in the published medical literature of unlicensed prescribing. These clinical audits, described below, can be used as guides by clinicians interested in prescribing in the given indications.

When prescribing unlicensed CBMPs, the foremost ethical consideration should always be “do no harm”, especially in treatment-resistant patients with highly debilitating diseases. Data available from clinical trials and RWE should be considered when prescribing THC based products in order to minimize patient harms.

### Personalising medicine

As evidence for the utility of minor cannabinoids and terpenes in medicinal cannabis products in improving patient outcomes builds (11), treatment can be tailored by the prescribing clinician to optimise response. This personalised medicine approach would currently not be feasible within RCTs and consequently RWE is more appropriate to unpick the full pharmacological armamentarium of cannabis. Results from such exploratory research can then be extended to develop more controlled research if given combinations of cannabis compounds appear to have superior efficacy to others for a particular indication.

### Botanical products

Botanical products, such as cannabis, contain a rich variety of substances within the product, including oils, carbohydrates, terpenoids, flavonoids, lipids, chlorophyll, amino acids, alkaloids, and fatty acids. Each one produces a different effect within the human body if consumed. These complex mixtures can contain hundred if not thousands of organic compounds of unknown concentration. These substances can work independently or in conjunction with one another, enhancing the bioavailability, pharmacokinetics, and pharmacodyamic effects of the active ingredient(s). Often, botanical drugs contain more than one active ingredient, and this can prove challenging when conducting pre-clinical assays that are geared at isolating the effects of independent compounds. This makes mechanism of action assays notoriously difficult as the effects could be attributable to a variety of compounds. Despite these challenges, botanical medicines can be developed in this way (12).

## What is real world evidence?

Real world evidence encompasses all forms of clinical data collected on patients outside of the traditional RCT setting. Far from being the “poorer cousin” to RCTs, RWE may be particularly valuable for researching novel applications for existing approved medications, or for building a body of research on products, such as medical cannabis, which do not naturally lend themselves to the traditional pharma research model. Typically, RWE begins after the phase I-III trials which bring a product to market. Once approved, RWE allows for pharmacovigilance data collection on adverse events and patient safety to continue, at lower costs, with larger numbers, in naturalistic conditions, and over an extended term, by applying appropriate analytics to the real-world use of medications (13). Such observational research, and its role in pharmacoepidemiology, is a fairly recent patient-centred approach to data (14).

### Real world evidence and technological advances

The landscape and boundaries around what constitutes RWE are already evolving. The creation of disease registries with the incorporation of wearable tech, biosensors, and other real time data collection methods, are beginning to benefit from the inclusion of even newer technologies, such as Artificial Intelligence (AI) and Natural Language Processing (NLP). Optical Character Recognition (OCR), and predictive and active modelling; technologies which have been pioneered in this context by companies such as Real World Health, Eleven Health, and Alta Flora. From this, we can understand the “bigger picture” surrounding a condition, shaping clinical care and allowing for the trial of novel interventions at lower cost, in ways which approximate how medicines are actually used, as opposed to the heavily supervised and standardised use of experimental medicines that is typical to RCTs. Such data can be integrated with that collated from other sources, such as Electronic Health Records (EHR), PRO data, biological assays, or even genomics (15–19).

### Real world evidence and rare diseases

Furthermore, many drug launches in recent years have found success by targeting either rare and genetic diseases, or in niche subpopulations of a wider disease–for example, defined by a distinct druggable target or biomarker (20). The most successful of such drug launches, in terms of favourable reimbursement decisions, are likely to be where these mitigate high unmet need (21). Preliminary RWE-based studies which combine RWD and analytical modules, including *via* machine learning algorithms, can help to identify those patient subgroups that have the worst outcomes, or are otherwise outliers in their disease burden: *“The specific study of real-world effectiveness in subgroups opens the possibility of research into effect modifiers (e.g., treatment by group interactions) and precision medicine”* (22). This in turn may improve our understanding of the variability in response, and to address concentrations of unmet need. Limitations of the non-randomised nature of treatment selection can be addressed by including comparison groups, or through the triangulation of multiple analytical approaches to improve confidence in inferred causal relationships.

### Real world evidence and inclusivity and diversity of patients

Another feature of RWE is its capability to reach beyond the “perfect” patients who typically inhabit RCTs; those who are stringently screened, recruited from within tight parameters, and almost always without comorbidities (23). A more inclusive recruitment, and procedural flexibility, allows for the personalisation of prescribing according to, and prioritising, patient need. Especially with regards to cannabis, a nuanced approach to prescribing is critical; individual sensitivities to THC and other cannabinoids are widely documented and are influenced not only by genetic factors and disease presentation, but also by any history of self-medication. Naïve patients, for example, typically require a more gradual titration of THC (24).

### Patient reported outcomes over time

Patient reported outcomes (PROs) are one of the most significant developments emerging from RWE. These have received immense investment from the US National Institutes of Health (NIH) and several new scales have been developed for this purpose. PRO measures are now required as elements of outcome measures for clinical trials funded by the NIH in the USA, to address a previously unmet need in the clinical research community (25). PROs put more emphasis on the patient’s quality of life and well-being and have been shown to be more sensitive to the effects of medical cannabis than traditional symptom-based measures. For example, a large and recent naturalistic German study on pain syndromes using PROs, found adding a CBMP significantly improved outcomes in neuropathic pain patients (26).

Furthermore, the European Medicines Agency (EMA) recently launched their Data Analysis and Real World Interrogation Network (DARWIN) (27) to deliver real world evidence on diseases, populations and the uses and performance of medicines, confirming the increasing understanding of the value of RWD. DARWIN aims to provide timely and reliable advice on the use, safety, and effectiveness of medicines for human use, from real world healthcare databases.

In the light of this growing call for RWE and PROs in 2022, NICE announced their intention to introduce RWE in NICE decision-making, with the objective to use RWD to address gaps in knowledge and to drive forward innovations for patients. Underlying this approach is the understanding of the value of RWE to improve health and social care delivery, patient health, and the effectiveness of intervention on patient outcomes in routine settings (28). The same standards should be applied to CBMPs.

Clearly, the RWE model is increasingly gaining traction within medical research. It both draws upon and encourages widespread stakeholder engagement and is less subjected to the financial constraints and incentives which shape pharmaceutical research presently. The benefits for cannabinoids research are clear–whole plant extracts with complex pharmacology and little potential for patent, fall outside the current model of drug development. Despite very promising initial data across a range of indications, barriers remain in response to a sparsity of high-quality evidence (29). Fortunately, there is an established pathway to prescription (most frequently *via* the private sector) meaning that RWE can be collected post-approval, in a way which still builds a much-needed evidence base to shape eventual expanded access. Such an approach facilitates and speeds up patient access, and provides greater equality of early access, as well as providing a complementary evidence base to RCTs.

These rapid developments in data resources and analytical techniques have been vital to assess and address the global COVID-19 crisis, and many guidelines are now beginning to include evidence from robust observational pharmacoepidemiological studies alongside RCTs (30).

## The value of real world evidence in relation to medical cannabis

Although these newer approaches could offer solutions to the lack of RCTs in relation to medical cannabis, here the acceptance of RWE is still not widespread. Reasons for this resistance have previously been described (31). The following sections outline RWE approaches that can be - and already are–used to generate scientific evidence on CBMPs.

### Prescribed cannabis in the United Kingdom: An exemplar of real world evidence

United Kingdom regulations stipulate that medicinal cannabis products, including those which are currently unlicensed, can be prescribed for any condition. To be eligible, a patient must be able to provide evidence of unsuccessful treatment episodes with conventional treatments and medical cannabis can only be prescribed by specialist physicians, not general practitioners. Despite being legal, medicinal cannabis products are not widely available through the NHS and eligible patients generally must seek–and pay for–treatment in the private sector.

International evidence indicates that large numbers of people seek treatment with medicinal cannabis once it has been legalised for medical use and, recognising the importance of generating evidence on the uses of and potential effectiveness of medicinal cannabis, Project Twenty21 (T21) was established as an observational registry to collect information on medicinal cannabis use in the United Kingdom and most recently set up in Australia as well. This study became operational in August 2020 and initial findings from the study are available (32, 33). By March 2022 data on the characteristics of people seeking treatment with medicinal cannabis were available for in excess of 2,000 individuals. Examination of these data highlight some unique advantages of real world evidence for studying the use of medically prescribed cannabis products including:

#### The number of different products used by individuals

In their simplest form RCTs typically compare differences in outcome between individuals receiving a specified dose of a single cannabis product and those receiving placebo. However, evidence from T21 indicates that individuals often receive multiple products concurrently while there are also considerable variations in doses: For example, among the 1,000+ people for who prescription data were available at 3 months, less than one third (31.7%) reported using a single product while 45.5% reported using two and 23.4% were using three or more prescribed cannabis products.

#### The length of treatment

Many trials examining the efficacy of prescribed cannabis products have used relatively short periods of treatment (34, 35). However, the nature of the conditions being treated and the experience of T21 patients indicates that, in practice these medications are often used long term. Specifically, just under 30% of the RCTs reported a follow up period of 7 days or less while only two studies reported following patients past 14 weeks (36). In contrast, by March 2022 T21 had 6 months follow-up data on 383 people with chronic pain with outcome monitoring ongoing.

#### The capacity to collect data on large samples

The two most common primary conditions within T21 are chronic pain and anxiety disorders: around 85% of all patients in T21 have one of these two categories of disorder and, in a relatively brief period of time we have been able to accumulate substantial numbers of individuals using medicinal cannabis to treat these conditions. For example, as of March 2022, three-month effectiveness data are available for 331 patients being treated for anxiety disorders. In comparison, the total number of participants in all RCTs of cannabis products to treat anxiety disorders, as identified in a recent systematic review and meta-analysis (37), was 54. Similarly, in a systematic review and meta-analysis of chronic pain, the indication for which medicinal cannabis has been most extensively studied, a total of 47 randomised controlled trails meeting their inclusion criteria were identified: together these studies enrolled a total of 4,271 individuals (approximately half of whom would have received placebo) (36). In contrast, T21 had data at treatment entry from a total of 1,176 patients by March 2022: this single study already has a sample size larger than any RCTs ever conducted, and the total number of patients exceeds 27% of the number of individuals ever studied within RCTs of medicinal cannabis for chronic pain.

#### Patients enrolled into RCTs do not represent the population of medicinal cannabis patients

For multiple reasons, RCTs have traditionally applied stringent exclusion criteria when recruiting patients. An initial assessment of the application of these criteria within the literature, and comparison with T21 patient characteristics indicates that the majority of real world medical cannabis patients would be excluded from these trials. For example:

#### Age and gender restrictions on patient recruitment

Findings from T21 indicate that seeking treatment with cannabis based medical products may be more common among males with 65% of T21 patients being male while there is a considerable range in ages. In contrast, while RCTs of pain appear evenly well balanced in both terms of age and gender it is notable that of the three RCTs examining the use of medicinal cannabis for anxiety disorders, one was based on an all male sample while a second focused on undergraduate university students and only one study included a sample likely to be representative of people seeking treatment for anxiety disorders in terms of both age and gender.

#### Prior or current use of cannabis

One exclusion criteria used across multiple RCTs is the exclusion of people currently using cannabis or with a (variously defined) past history of cannabis use. However, unsurprisingly, T21 data indicate that the majority of patients seeking treatment with CBMPs have some current or prior experience with it: 88.5% reporting some prior experience and 65.1% reporting that they were currently using it (the majority reported using it for medicinal purposes).

#### The use of other pharmaceuticals

Another exclusion criteria reported in the literature involves the exclusion of people currently using other (non-cannabis) prescribed pharmaceuticals. Again, however, T21 data indicate that such use is common: 71% reported that they were currently using other prescribed medications with the highest prevalence of any prescription drug using occurring among chronic patients (80.5% were using other prescribed medications) who were using a mean number of 4.1 different medications.

#### Comorbid illness

A common exclusion criteria across trials is the exclusion of individuals with comorbid physical or psychiatric conditions. However, many if not the majority of people presenting for treatment for a specific condition will have comorbid pathology and limiting recruitment to only “pure” cases of a specific disorder risks generating safety and efficacy data that would be uninformative about the likely safety and effectiveness of a treatment within the population of people seeking treatment for that condition. For example, among the three most common primary conditions within T21: 58.7% of chronic pain patients would potentially be excluded on the basis of comorbid pathologies. Similarly, 89.2% of people seeking treatment for anxiety disorders and 92.9% of those seeking treatment for PTSD reported being diagnosed with a comorbid psychiatric condition. Arguably, these may be underestimates of the percentages of the T21 sample who may be ineligible to participate in a trial of treatment for that condition: we have not calculated the percentage of people with one category of pain or anxiety disorder who meet criteria for multiple such disorders (e.g., people with arthritic pain who also experience fibromyalgia; people with generalised anxiety disorder who also have social phobia) although such comorbidities may be common. Nor have we screened individuals which may potentially reveal additional diagnoses.

#### Cost

As highlighted previously RCTs are very costly (9), and in comparison, RWE can provide data at much less cost. For example, we estimate that the total costs associated with the curation of data from T21 would be insufficient to support a single randomised trial of 100 patients.

Together, this evidence indicates that there are likely to be large differences between the way that medicinal cannabis is delivered in the real world and the ways in which it has been studied in traditional trials. Therefore, there are serious concerns about the utility of evidence derived from RCTs and the extent to which findings concerning both the safety and efficacy of prescribed cannabis may be applicable to the real world.

## Utilising the power of individual clinical interventions or *N* = 1 trials

*N* = 1 trials are the core of medical practice since every time a medicine is prescribed an *n* = 1 experiment is being conducted. In some patients the experiment works and in others it fails, the patient either does not respond, or the adverse effects outweigh the therapeutic benefit. One might therefore expect that doctors would welcome patients who have conducted successful self-treatment with cannabis since it is almost certain that prescribing medical cannabis to these individuals will work, providing a therapeutic guarantee for both patient and prescriber.

The resurrection of CBMPs following its banning by the United Nations Conventions is partly attributable to *n* = 1 trials conducted in children with intractable epilepsy. The first patient was Charlotte Figi in the USA who inspired UK parents of children with similar epilepsies, notably Alfie Dingley and Billy Caldwell (38). These children were facing death and/or brain damage from multiple seizures resistant to licenced treatments and CBPMs restored them to close to normality and also allowed them to reduce, and in some cases cease, use of other medicines, as outlined in the Drug Science audits below. In the case of Billy, the proof of therapeutic efficacy was established by the confiscation of his medical cannabis by UK customs which led to a life-threating episode of status epilepticus requiring admission to intensive care. The public outcry over such callous treatment by the UK government was one reason for the rescheduling of medical cannabis in November 2018.

In scientific terms Billy was a “natural experiment”. Such a natural experiment shares many features, including its analytic approach, with a traditional ABA(B) experimental trial, which remains one of the most powerful methodologies for examining a medical intervention.

The value of natural experiments has been explored particularly for studying developmental psychopathology and the case of Billy Caldwell exemplifies how a “naturally occurring” (albeit arguably unethical) change in environment can be exploited to examine the effects of that changed environment (access to CBMPs). The UK government accepted that in these cases CBMPs worked.

An ABA(B) trial design is well suited for determining whether medical cannabis is efficacious. Bayesian analysis can also combine separate ABA(B) results from different populations of patients, such as those who consume medical cannabis and non-cannabis users, stratified as suggested by experts whose experience has identified possible confounding variables (39). This approach is known as multilevel regression and post-stratification (40).

## Bayesian analysis

**FIGURE 1:**
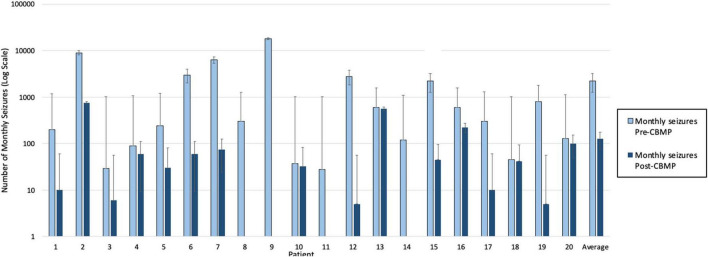
Monthly seizure frequency pre- and post- cannabis based medicinal products (CBMPs).

In each of the two studies data was collected from children who were 18 years old or younger, all suffering from intractable epilepsy (41, 42). Treatment started at the point consent was granted, but after the study team found the seizures of the participants were substantially reduced, they analysed the data, which showed an average reduction in monthly seizures of 86% across the cohort (please see Figure 1).

To see how this descriptive statistic might extend to more patients, a Bayesian analysis established a probability that an 11th patient would experience a reduction in seizures. The analysis started with the assumption that any percentage reduction from 0 to 100% was equally likely (the prior probability), incorporated the 10 successes and no failures to improve, which gave a 0.92 probability (99% credible interval of 0.66–1.00) that the next patient would experience a reduction (the posterior probability).

The study team then continued to collect more data, which again showed substantial improvement for every participant, 10 more successes and no failures, data used to revise the posterior probability from the previous 10 participants, resulting in a 0.95 probability (credible interval of 0.82–1.00). The additional 10 increased the probability by only 0.03, but the credibility of the 0.95 is greater than for the 0.92.

The Bayesian result provides exactly what prescribing physicians want to know: what is the probability that this patient will benefit from this treatment, and how certain they can be about that probability. The only remaining uncertainty is whether the next patient is from the same population of patients as those already observed. Causality was well established, by the 20 patients of above studies (41, 42) and so far, no confounding variables are evident in those studies.

Looking at inhaled cannabis for chronic neuropathic pain, an earlier Bayesian hierarchical model allowed the synthesis of all available patient data from five RCTs with disparate design and outcome reporting. Inhaled cannabis appears to provide short-term relief from chronic neuropathic pain for one in five to six patients treated (43).

Such observational studies are best analysed within a Bayesian framework, as findings are expressions of the authors’ uncertainty about the results, so randomisation is irrelevant, and data can be analysed just as they would with randomised studies (44).

However, the Bayesian framework can also incorporate analyses of various form of bias in RWE studies, though this was not done in above studies (41, 42) because all patients put forward by their parents were included, the demographics were reported, and the authors were unaware of any bias that might prevent generalising the results to the population of similarly chosen child patients. It is difficult to see how any bias of the authors could so substantially affect such an intractable disease state. Indeed, the Bayesian analysis started with a uniform prior distribution: the population proportion of patients experiencing a reduction in seizures was equally likely to be anywhere between 0 and 100 percent.

## Case study research

Case studies provide in-depth insight into the holistic impact of medicinal cannabis treatment on an individual or a cohort. This approach provides a deep understanding of how CBPMs are administered to enable patients to meet their treatment goals. Case studies support the development of clinical expertise and reduce stigmatisation by spotlighting novel approaches in clinical prescribing, thus legitimising the efforts for medical development in a given indication with a given intervention. Published case studies have demonstrated where medicinal cannabis prescription has reduced the need for hospitalisation, emergency treatment and other prescription medications, thus collectively offering cost savings to the NHS and other health service providers (36). Despite this, case studies highlight the significant and often prohibitive cost of private treatment to the individual.

Rare diseases pose a unique challenge to research as the population affected can be too small (41, 42) or there may be insufficient funding to facilitate an RCT. Medicinal cannabis has established considerable benefit in conditions with no other licenced treatments, such as in rare syndromes underlying childhood epilepsy (41, 42), Ehlers Danlos Syndrome (45) and also in conditions with poor treatment outcomes such as lung cancer (please see Box 2). Therefore, case studies provide evidence where medicinal cannabis provides a novel approach to treatment in difficult to treat conditions.

**BOX 2. Case Study Ehlers-Danlos Syndrome (45).**

Hypermobile Ehlers-Danlos syndrome (EDS) is a multisystemic condition comprising a range of symptoms including joint hypermobility, joint pain, visceral, and autonomic dysfunction. Delayed diagnosis results in this relatively young cohort of patients presenting late, with several complications and having exhausted standard treatment. Symptom alleviation provided by CBMPs has the potential to provide improved care and quality of life for these patients. The patient experienced symptoms including finger, jaw, dual hip, and shoulder dislocations, autonomic dysfunction, postural hypotension, gastroparesis requiring parenteral nutrition, and bladder spasms requiring catheterisation. The patient also required a microdiscectomy. Her pharmacological treatment of pain accumulated a minimum morphine equivalent daily dose of 220 mg. Within 3 months of medicinal cannabis treatment, her pain levels had considerably subsided, and she experienced a reduction in muscular spasms. After 6 months of treatment, she was able to cease all opioid medications, no longer needed to self-catheterise or receive parenteral nutrition. Following treatment, her Barthel Index score improved from 15 (totally dependent) to 100 (independent). Socioeconomic health variables associated with treatment showed a significant reduction in A&E and hospital admissions and formal NHS-funded care. She was able to return to full-time education and part-time work. The patient is unable to obtain an NHS prescription for medicinal cannabis. Therefore, she has resorted to self-medicating with illegal cannabis, risking product contamination and criminalisation. She is now receiving a subsidised private prescription of unlicensed whole-plant CBMPs through Project Twenty21, at a cost of £450 a month. This includes a balanced CBD: THC sublingual oil and high CBD sublingual oil three times daily, in addition to inhaled high THC cannabis flower for acute symptom relief as necessary. Barriers to access in this case included insufficient clinician knowledge and education of medicinal cannabis, restrictive guidelines, fears of adverse effects, and cost and supply issues. This case report demonstrates the need for increased awareness and clinical expertise of this treatment option and calls for the expansion of clinical data collection of the therapeutic and economic benefits of medical cannabis.

## Basket protocols

“Basket” protocols describe the approach wherein one specific intervention i.e., medical cannabis, is given across several disorders (46). Under such a protocol, variable dosing, frequency of dosing and the integration of digital platforms to collect real-time patient data could be useful in deeply phenotyping treatment interventions which is not common practice under conventional RCTs (46). Further, basket protocols lend themselves to medical cannabis in that current knowledge of the symptomatic effects of medical cannabis are broad and far reaching and changes in quality of life are amongst the most reported treatment outcome (see Project Twenty 21). In chronic illness there are several overlapping clinical symptoms clustering around the physical; pain, insomnia, inflammation and neuropsychiatric; depression, anxiety, and anhedonia (47).

Conventional RCTs have disorder specific primary endpoints e.g., a change in pain scales for individuals with chronic pain, however, a more encompassing set of primary outcomes including the aforementioned physical and neuropsychiatric sequelae in addition to quality of life and wellbeing scales would allow for a more holistic view of the *trans*-therapeutic efficacy of medical cannabis. These *trans*-diagnostic approaches to capturing endpoints and including patients would better capture the multidimensional breadth of the clinical utility of medical cannabis as opposed to restricting it to *a priori* singular endpoints which do not best capture its effectiveness. Fundamentally, such a method will allow for a greater understanding of the best approach to take with these new medicines, ensuring their clinical utility reaches as many patients as possible (46).

## Citizen science

Citizen science initiatives could also provide valuable data, specifically in the context of medical cannabis. Restrictive drug policies make placebo-controlled studies on CBMPs difficult and expensive to conduct in laboratory settings due to the need for home office licences and intensive regulatory oversight. Citizen-science approaches may help to overcome these challenges (48). Citizen science involves individuals implementing their own placebo control at home, using their own acquired medical cannabis products, following online instructions to conduct self experimentation over a given time period with self-reported clinical scales to assess the value of the intervention. The advantages are the low cost and the ability to recruit participants globally (48).

This method could be pragmatically useful due to the legalities and issues surrounding access to medical cannabis and the lack of access to institutional research funding with medical cannabis in the UK. Citizen science initiatives allow participants to record changes in their own physical and mental wellbeing, these can be assessed through integrating clinical questionnaires onto digital apps, such as ReLeaf (49), and through objective biometric testing through devices such as FitBits and Oura rings. Hence, a rich set of data can be acquired through vastly larger numbers of individuals than would be possible in an RCT setting–at a fraction of the price.

The results from such studies could lead to more defined hypothesis generation based on real-world data, thereby reducing risk in the drug development process and saving valuable economic resources.

## Limitations of using real world evidence and ways of mitigating them

Despite these numerous advantages, there are also several limitations to the use of RWE. Many of these were instrumental in the development and elevation of RCTs and include limitations related to a potential inability to adequately control for potential confounding associated with treatment assignment that is traditionally addressed through randomization. Missing data is common as the collection of specific measures is not under the control of the researchers.

The availability of great amounts of data from large samples (sometimes in millions) may increase risks of type I errors, although this can be overcome from careful pre-specification of analysis protocols. This is less relevant to real world studies of cannabis that assess *a priori* hypotheses about the effectiveness in specific conditions.

Large scale real world data sets such as insurance or electronic health records (EHR) databases often lack refined outcome assessments and therefore focus on clearly defined endpoints such as mortality. Such a focus may be ill suited to assessing the effectiveness of CBMPs as some of most robust benefits of cannabis appear to be in symptom reduction and improvements in quality of life. Nonetheless, it is possible to overcome this potential limitation by incorporating patients reported outcome measures into real world data collections.

Finally, it is the case that RWE is not as yet, accepted by regulatory authorities as a basis for licencing medications. However, NICE’s recent development of an RWE framework is an indication that this *status quo* might be changing. Within this framework, NICE also explores how any potential bias related to RWE can best be assessed and addressed (28). Box 3 sums up our key recommendations for implementing RWE in relation to CBMPs.

**BOX 3. 12 Key recommendations.**

1. Cannabis has an excellent safety profile and is an established medicine. Concerns about the perceived lack of RCT evidence are misplaced as many patient-centred approaches can be, and have already been, applied.
2. RWE approaches are the key to accelerate development of clinical effectiveness evidence on CBMPs across a wide range of disorders, so we need to move away from the current focus on RCTs and incorporate RWE results. Patient numbers in RWE for medical cannabis are already much larger and have greater temporal sensitivity (due to ongoing longitudinal data acquisition) than all RCTs to date combined.
3. RWE can provide data for specific patients that RCT results cannot. The reality of medicine is that for every patient every new treatment is an *n* = 1 experiment. Individual patient outcome measures are the gold standard of the value of the treatment.
4. RWE provides more ecologically valid data as it can be acquired from a much larger range of patients than RCTs. This is because RCTs usually exclude people with co-morbid conditions, despite such patients being the majority presenting in clinical practice.
5. There is growing consensus amongst practitioners and regulators that RWE is essential to improve the ecological validity of the broader utility and clinical outcomes of new medicines. These advances would greatly help to optimise personal treatment protocols, supporting a move to personalised and precision based medicine- a key goal in 21st century medical practice.
6. The historic predominance of WEIRD (Western, Educated, Industrialized, Rich, and Democratic) participants in western medicine RCTs means their results are not representative of the general population. Basing efficacy on such a subpopulation leads to ethnic and racial disparities in healthcare. This can be actively combatted through the acquisition of more representative data acquired from the real world (50).
7. The European Medicines Agency (EMA) has recently launched their Data Analysis and Real World Interrogation Network (DARWIN EU) to deliver real world evidence on diseases, populations and the uses and performance of medicines, confirming the increasing understanding of the value of RWD.
8. RWE can address the need for more data to develop the current scientific evidence base. So far, there is no homogenous way of data collection on medical cannabis patients and the number of prescriptions written across countries. In Canada, the development of a large-scale database allows for side-effects to be monitored and managed more effectively (51). Results can then be incorporated to develop regulation and policy-making.
9. The available RWE evidence of CBMPs highlight their benefits in various clinical conditions. Specially for treatment-resistant patients and in selected medical conditions, CBMPs can offer an important therapeutic option.
10. PROs matter. GMC guidance on good medical practice makes it clear that all registered doctors must take into account and respect patients’ views and experience. Ideally, doctors should develop the evidence base together with their patients to better define indications. Areas which have significant data gaps will still require more rigorous studies and RCTs.
11. The collection of safety data is essential. Doctors and other health care providers need to be able to monitor the outcome of any treatment. Adverse effects must be registered and addressed, e.g., through the yellow card system in the UK or through a medicine-specific database.
12. Pharmacovigilance will remain important. Any harms need to be reported. Particularly attention should be paid to medical cannabis prescription and dependence. Specific medical cannabis dependence questionnaires have been developed and should be included in clinical pharmacovigilance.

## Conclusion

Over a million UK patients are self-medicating with illicit cannabis products. The international database evidence suggests that these drugs offer a notable advantage in treatment for many patients in whom current medicines are either ineffective or poorly tolerated. Present findings from RWE globally are highly suggestive of a pattern of evidence which deserves a level of recognition it does not currently receive.

The criticism of the lack of placebo-controlled trials is misplaced. Prescribers often mistakenly state that without these they cannot prescribe (31). However, there are over 50 medicines or indications that have been licensed by Food and Drug Administration and/or European Medicines Agency between 1999 and 2014 without RCT data (52).

Statements such as “insufficient evidence of efficacy” are common and used even in the face of strong personal evidence from patients that CBPMs work and, in many cases, can be life-changing and well tolerated. Many doctors fail to include the evidence of the patient’s lived experience and cite the lack of placebo-controlled trials (for most indications) for their hesitation to prescribe (31). The failure of the medical and pharmacy professions to embrace CBMPs despite a legal change nearly 4 years ago allowing their prescription, is clearly detrimental to patients.

Cannabis has an excellent safety profile and is a historically established medicine (1). Pragmatic long-term studies (such as T21) can further confirm its safety and effectiveness. RWE is the best way to get clinically useful data on medical cannabis. In addition to controlled clinical research it is imperative to supplement such efforts with studies that assess the impacts of cannabis medicine in the real-world and outside of a laboratory setting.

We hope that this paper will aid policymakers and prescribers understand the value of RWE in relation to medical cannabis and help them develop approaches to overcome the current situation, which is ultimately harmful to patients, restricting access to medicines that could bring relief.

## Author contributions

AS developed the initial manuscript. RZ wrote the sections on medical cannabis and epilepsy, as well as novel approaches to RWE. ML and AA-F contributed sections on Project Twenty21. LP contributed sections on utilising the power of individual clinical interventions or *N* = 1 trials and Bayesian analysis. DN had overview of the work. All authors were involved in revising the manuscript and all agreed on the final draft to be submitted.

## Abbreviations

AI: Artificial Intelligence
BPNA: British Paediatric Neurology Association
CBD: Cannabidiol
CBMPs: Cannabis based medicinal products
DARWIN EU: Data Analysis and Real World Interrogation Network
THC: Delta-9-tetrahydrocannabinol
EHR: Electronic Health Records
EMA: European Medicines Agency
FDA: Food and Drug Administration
GMC: General Medical Council
HCPs: Health Care Providers
MHRA: Medicines and Healthcare Regulatory Agency
MS: Multiple Sclerosis
NHS: National Health Service
NIH: National Institutes of Health
NICE: National Institute for Health and Care Excellence
NLP: Natural Language Processing
OCR: Optical Character Recognition
PROs: Patient reported outcomes
RCTs: Randomised Controlled Trials
RWE: Real World Evidence
RWD: Real World Data
T21: Project Twenty21
WEIRD: Western Educated, Industrialized Rich and Democratic.
